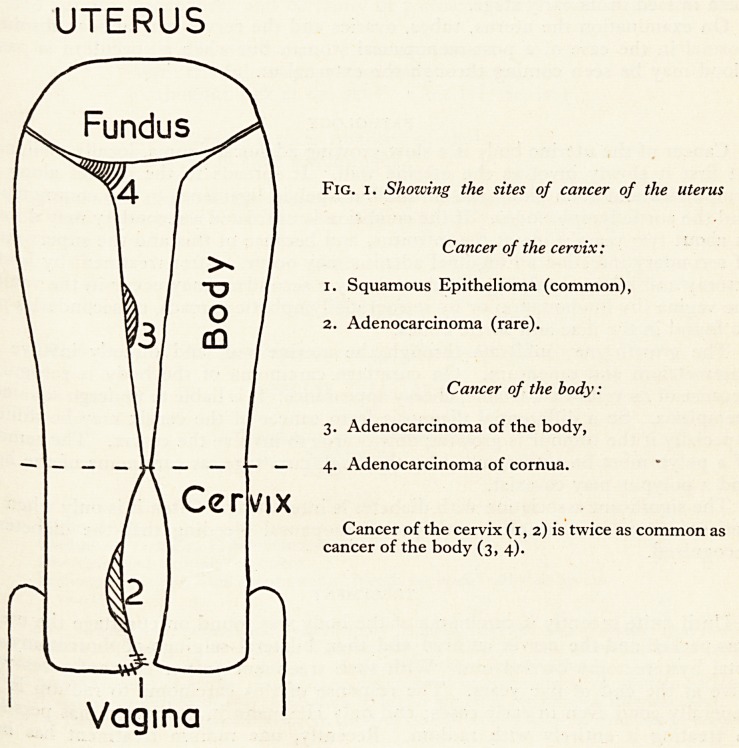# Cancer of the Uterus

**Published:** 1956-01

**Authors:** G. Gordon Lennon

**Affiliations:** Professor of Obstetrics and Gynaecology, University of Bristol


### CANCER OF THE UTERUS

BY

G. GORDON LENNON, CH.M., F.R.C.O.G., M.M.S.A.
*Professor of Obstetrics and Gynaecology, University of Bristol*

Apart from sarcoma of the uterus and chorionepithelioma which are both extremely rare, malignant disease of the uterus may be considered under two headings, cancer of the cervix and cancer of the body. In Bristol in 1950 cancer of the uterus accounted for 10 per cent. of female cancer deaths. In comparison breast cancer caused 17.4 per cent. of female cancer deaths.

### CANCER OF THE CERVIX

The general practitioner can help us greatly in the treatment of cancer of the cervix by referring cases earlier. Here are two examples:

A married woman, aged 41, who was seen by me at Oxford one October had been to her doctor in April the same year with irregular menstruation. She had been treated by rest and Stilboestrol, and no pelvic examination had been done. She was a busy woman, owner of a café by the river, and dependent largely on the summer trade for her living. Seven months later the same doctor saw her again and on pelvic examination was horrified to find unmistakable signs of cancer of the cervix.

A married woman, aged 31, referred to me by a Bristol practitioner had a complaint of irregular menstrual loss and contact bleeding. The doctor had passed a speculum and had diagnosed cancer of the cervix before referring her to out-patients'. The cancer was still confined to the cervix and I treated her with radium followed by Wertheim hysterectomy and pelvic lymphadenectomy. There was no evidence of tumour in the excised lymph nodes. This woman is well with no sign of recurrence nearly two years afterwards. I dare to hope she may be cured.

*Better far to feel with a finger or see with a speculum than be humiliated by a hormone!*

Of course, many patients delay seeking medical advice, and doctors cannot be held responsible for this. On the other hand, a few cases progress so rapidly that in spite of early diagnosis not much can be done, for example:

A married woman, aged 31, whom I saw recently had had symptoms for only 3 months. The complaint was of pain down the left leg and in the left groin. This patient had extension of cancer of the cervix out to the lateral pelvic wall, involving the left obturator nerve, and also blocking the left ureter so that the kidney on that side had ceased to function. (Pain as an initial symptom occurs in only 2 per cent. of cases (Way, 1951). It is usually a late symptom of untreated cases indicating involvement of nerves or the presence of bone secondaries.)

### DURATION OF SYMPTOMS

Three months is a relatively short delay: the time-lag is often much worse as can be seen from the Report of the South-Western Regional Cancer Records Bureau, 1950 (see Table I).

TABLE I

TIME-LAG BETWEEN FIRST SYMPTOMS AND TREATMENT

| Site | 3 months or under (per cent.) | 3–12 months (per cent.) | Over 1 year (per cent.) | Over 2 years (per cent.) |
|---|---|---|---|---|
| Uterus | 34 | 28 | 14 | 24 |
| Breast | 38 | 30 | 10 | 22 |
| Lip and Mouth | 24 | 28 | 20 | 28 |

Probably in more than 50 per cent. of cases the time-lag is the patient's own responsibility (Way, 1951), for such obvious reasons as onset of menopause, fear, and even erroneous neighbourly advice. Many give no reason for the delay. May I suggest that more efficient, more frank education about the menstrual function, probably at school (*not* " cancer education ") will help greatly to obviate delay in seeking medical advice when menstrual irregularities occur?

But more important, there is medical delay. Of 56 cases Way states that 25 consulted their doctor within one month, yet only 12 were sent to hospital at once, and one other refused to go for a year. The remaining 12 were not referred until they had had symptoms for 3–17 months, and in none was vaginal examination carried out. An important rule for the general practitioner is:
*Do not wait for the bleeding to stop before passing the speculum.*

### SYMPTOMS

In 90 per cent. of cases the primary complaint is vaginal haemorrhage or blood-stained vaginal discharge, especially after coitus, douching, micturition, or vaginal examination.

### AGE INCIDENCE

Eighty-five per cent. of the cases are between 40 and 70 years of age. The youngest I have seen myself was aged 24. Thus, it cannot be too strongly emphasized that irregular vaginal bleeding occurring after the age of 40 is strongly suggestive of carcinoma of the cervix, and should be regarded as due to this until proved otherwise. The proof will be obtained by examination of the cervix with a speculum and, if necessary, by biopsy and histological examination.

### SIGNS

In the early stages the cervix will show erosion with raised everted edges. It will bleed probably on examination or certainly when touched. In the more advanced cases parts of the growth will break off and the vaginal discharge will be not only haemorrhagic but offensive as well because of superimposed infection. The growth will feel hard and irregular. Later there may be either a cauliflower growth coming down the vagina from the cervix or cavitation due to an ulcerative process and the cervix will be almost completely absent. There will also be fixity of the uterus due either to the growth invading the lateral ligaments or to infection. Secondaries in bone are late and are usually sacral and vertebral.

It is common for authors of textbooks to make a great point of the clinical staging of cases of carcinoma of the cervix e.g.:

Stage I. Tumour limited to cervix.

Stage II. Tumour involves vaginal vault and/or one or both parametria but no fixity.

Stage III. Tumour involves one or both parametria and growth is fixed to pelvic wall, or tumour encroaches on lower third of vagina.

Stage IV. Tumour invades bladder or rectum or there are metastases outside pelvis.

Unless the same person is doing it all the time this clinical staging (under anaesthesia) is very difficult to assess accurately. Even cases labelled Stage I may be in fact Stage IV, for example, there may appear to be a Stage I growth but the patient also has haematuria indicating bladder involvement. At a cancer conference in Newcastle some years ago Way invited four well-known gynaecologists to ' stage " a case of carcinoma and he found that each gynaecologist put it into a differ nt stage. This may account for differences in results reported from different centres.

### PATHOLOGY

In most cases cancer of the cervix is a squamous-celled growth arising from the junction of the squamous epithelium with the columnar epithelium at the external

*os.* Adenocarcinoma of the cervix is rare and arises in the region of the cervical canal; it is more malignant and less amenable to radiation therapy (see Fig. 1).

The spread of cancer of the cervix is directly from the cervix on to the vaginal fornices and in the late stages into the bladder (vesico-vaginal fistula) and into the rectum (recto-vaginal fistula). Spread also occurs along the lymphatics into the lateral aspect of the supra-vaginal portion of the cervix (Mackenrodt's ligaments), out to the lymph nodes in the obturator canal and along the internal iliac artery and from there upwards to the external iliac nodes, and along the common iliac chain to the aortic nodes. Also there is spread along the utero-sacral ligaments backwards on either side of the rectum to the region of the third piece of sacrum. Pain may be present for a considerable time before x-rays show the secondary growth in the sacral or lumbar vertebrae. In one case I waited nine months after the onset of pain before demonstrating by radiography a secondary in a lumbar vertebra. The type of carcinoma one likes to see is the one that comes to meet you as a proliferative growth down the vagina. Usually this type melts away with radium treatment whereas the ulcerative type is more resistant. Unfortunately, the ulcerative type is rather more common than the hypertrophic type. The cause of death in cases of cancer of the cervix is usually uraemia due to pyelonephritis or obstruction of the ureters. Occasionally cases die from intestinal obstruction. In the late stages there is a profound degree of anaemia and gross fatigue from intense bone pain. The intestinal obstruction and blockage of ureters is not necessarily always due to extension of growth but may result from fibrosis due to radiotherapy or surgical trauma.

Occasionally, carcinoma occurs in the stump of a cervix which has been left after sub-total hysterectomy.

A married woman aged 59 was referred to me by her doctor in September 1951. His letter was so good that I reproduce it in full: "This patient had a subtotal hysterectomy performed in 1939. She came to consult me about her blood pressure and happened to mention that at Christmas last she had seen a little blood P.V. and again once since. No loss of weight or bladder symptoms and no vaginal discharge. On examination I found a large fleshy mass growing from the cervix. It bled very easily and a piece of necrotic material broke away. No glands palpable. Her blood pressure was 160/100."

This woman had had a sub-total hysterectomy performed 10 years previously for uterine fibroids. Biopsy of a friable fragment from the cervix showed papilliform adenocarcinoma. The patient was given radium treatment and a fortnight later I performed a Wertheim hysterectomy. In so far as I could I excised the stump of the cervix and also performed pelvic lymphadenectomy. The pathologist's report was as follows:

"The excised cervical stump is infiltrated by glandular carcinoma which spreads upwards through two-thirds of its substance, and laterally into the vaginal vault: there is, however, a narrow belt of normal vaginal mucosa around it. Glands dissected out from the right and left iliac groups show only non-specific adenitis; those from the right and left obturator groups are heavily infiltrated with more anaplastic spheroidal-cell secondary growth." (Dr. A. Taylor.)

This patient made an uninterrupted recovery and doctors may recall seeing her at a recent clinical meeting. Three years later there is no evidence of recurrence and she is very fit and well.

It is said that radium therapy only gives a 25 per cent. five-year cure rate in stump cancer (Redman, 1952). Of course, the best treatment is to prevent it by performing total hysterectomy in the first instance. One hopes that in future the operation of sub-total hysterectomy will not be performed. Nowadays total hysterectomy carries no more risk than sub-total hysterectomy.

### RECENT ADVANCES IN PATHOLOGY

The Americans have recently stressed the clinical stage " O " which they call *cancer in situ* or pre-clinical cancer. The best definition I know of this is that given by Hertig (1952):

" It would appear, therefore, on the basis of its incidence, age distribution, selective racial incidence, biologic behaviour, histologic appearance, and associated exfoliative cytology, that carcinoma *in situ* is the preinvasive stage of squamous carcinoma of the cervix."

This condition is one for the pathologists to argue about when a biopsy is taken from a suspected cervix. In this connexion I would utter a word of warning.

A married woman, aged 36, who had a young child and was anxious for other children, was seen by a gynaecologist; he was a little worried about an erosion of her cervix and performed a biopsy. The report was "? *carcinoma in situ*" and upon this finding the poor woman had her uterus, tubes and ovaries removed. There had been some technical difficulty at the operation and ten days later she was found to be wet all the time. She had, in fact, a traumatic vesico-vaginal fistula and it was only at this stage that I saw her with a request to close her fistula. This was done with satisfactory healing and she is now well. I find it hard to believe that it was necessary to remove this uterus in the first place. The condition should have been watched and further biopsy performed at a later date.

A good deal has been done in America and a little in this country on the " screening " of gynaecological patients for carcinoma of the cervix and the body of the uterus by means of the examination of vaginal smears. This is highly specialized work and the technicians and pathologists have to be trained over a long time in order to recognize the type of cells exfoliated. Recently, it has been indicated that perhaps the cost of such investigations and the time involved does not justify their introduction as a routine measure.

### TREATMENT

Until the end of the last century all that could be advised for women with carcinoma of the cervix was douching to relieve the vaginal discharge and hot cautery for proliferative masses. From 1900 onwards, after the introduction of Wertheim hysterectomy, attempts were made to remove the offending growth. The primary mortality of this operation even in the best hands was 14 per cent. and the incidence of ureteral fistual was 12 per cent. (Bonney 1941). Consequently, when radium treatment was introduced about 1920 it was found to be so much easier to carry out that there was a complete swing away from surgical treatment—except by Victor Bonney. He continued to practice Wertheim hysterectomy and was in a position a few years ago to review a series of 500 cases. Of these cases he claimed 159 alive after 5 years out of 300 in which lymphatic glands were not involved, and 42 alive after 5 years out of 200 where the lymphatic glands were involved. Bonney inclined to the view that lymphatic gland involvement doubled the operation risk and more than halved the ten-year cure rate (1949).

Until quite recently all cases have been treated with radium either in one large continuous dose (Paris method) or in divided doses (Stockholm method). When all cases are treated thus the overall five-year cure rate is about 35 per cent. Treatment of Stage I cases by radium will give a five-year cure rate in about 62 per cent. of the cases, perhaps even slightly more (Stockholm, 1954).

In the South-Western Region (1950) the five-year cure rate for Stage I is given as 41.1 per cent. This is a very poor figure and either the staging was inaccurate or treatment was not as efficient as it might have been.

### RECENT ADVANCES IN TREATMENT

Even Stage I carcinoma of the cervix may already have microscopic involvement of the lymph nodes. This is stated to occur in about 20 per cent. of Stage I cases (Read, 1948). Recently, therefore, there has been a combined radium and surgery approach. Patients are given part of the full radium dosage and are then reviewed; if they are considered suitable cases Wertheim hysterectomy and pelvic lymphadenectomy are carried out a fortnight after the last radium treatment. If they are rejected for operative attack, the radium dosage is then completed. Modern conditions (better anaesthesia, blood transfusion, &c.) have made the operation a much less hazardous one. Several series of cases have already been reported; thus in Boston the operative mortality in 473 cases was 1.7 per cent. (Meigs, 1955), and in Leeds there were no deaths in 103 cases (Currie, 1952). Of course, surgery will only be possible in about 20 per cent. of cases, i.e. those with no contra-indication to major surgery, in Stages I, II, and a few in Stage III. Biopsy indicating little response to radium will tend to extend the indication for Wertheim hysterectomy in a case that might otherwise be rejected (Glucksmann, 1949). In the period 1951–1954 I have performed surgery on 47 per cent. of the cases seen.

Extended surgical procedures for advanced cases have been introduced, particularly by Brunschwig in New York. The vagina, uterus, tubes, ovaries, related lymph nodes, and bladder may be removed, and the ureters implanted in the bowel (" North America " operation). The vagina, uterus, tubes, ovaries, related lymph nodes, and rectum can be removed, and a colostomy performed (" South America " operation). Total pelvic exenteration means the provison of a " wet " (urine) colostomy, and removal of rectum, bladder, vagina, uterus, tubes, ovaries and related lymph nodes. These are, in truth, extensive procedures, not to be undertaken lightly, and, indeed, the question may be posed, " Are they, in fact, worth while? "

High-voltage therapy is used mostly to attack the lymph nodes in inoperable cases, or in recurrences.

### CANCER OF THE BODY

This cancer occurs in the cavity of the uterus or in the cornua (Fig. 1). In the cornua it may be missed on curettage. This adenocarcinoma of the uterine cavity is rarer than carcinoma of the cervix but it is the most important cause, after

FIG. 1. *Showing the sites of cancer of the uterus*

*Cancer of the cervix:*

1. Squamous Epithelioma (common),
2. Adenocarcinoma (rare).

*Cancer of the body:*

3. Adenocarcinoma of the body,
4. Adenocarcinoma of cornua.

Cancer of the cervix (1, 2) is twice as common as cancer of the body (3, 4).

cancer of the cervix, of post-menopausal bleeding. It has been stated that if cancer of the cervix is the uterine cancer of the childbearing woman, cancer of the body is the uterine cancer of the nullipara, and its association with the diabetic woman is statistically significant (Way, 1954). It is comparatively rare before the age of 50 but I have seen it in a woman of 28.

It has been pointed out that the woman with menopausal menorrhagia is three and one half times more likely than the normal woman to develop cancer of the body of the uterus (Randall, 1946). Every case of menopausal menorrhagia must be curetted to exclude it. Indeed, every woman of 40 or over with menstrual irregularity must be curetted. This is a major argument apart from many others for hysterectomy rather than radium to procure the menopause. It is a Bristol graduate —Green-Armytage (1950)—who has urged the use of vaginal hysterectomy for menopausal menorrhagia.

### CLINICAL FEATURES

The typical history is bleeding in a woman over 50 who has had no periods for a few years and who, although perhaps married, has never had any children. While post menopausal bleeding may be due to other causes it is most important to exclude cancer of the body before anything else. Let me emphasize once again the exclusion of this cause in every woman of 40 or over who has irregular menstruation. I have had one such case this year in a woman aged 47. If this had been ascribed to the menopause and no curettage performed this carcinoma would have been missed in its early stage.

On examination the uterus, tubes, ovaries and the cervix may appear absolutely normal in the case of a post-menopausal woman but when a speculum is passed blood may be seen coming through the external *os*.

### PATHOLOGY

Cancer of the uterine body is a slow-growing adenocarcinoma, locally malignant. At first it slowly involves the uterine wall. It spreads to the ovaries along the lymphatics and then along the infundibulo-pelvic ligaments to the common iliac and the aortic lymph nodes. If the condition is untreated a secondary may develop in about two years' time at the introitus, and because of this and the supervention of secondary infection an inguinal adenitis may occur. After treatment by hysterectomy and bilateral salpingo-oöphorectomy a secondary may occur in the vault of the vagina (by implantation or by retrograde lymphatic spread), or secondaries may be found in the iliac and aortic nodes.

The growth may infiltrate through the uterine wall and directly involve the parametrium and omentum. On curettage carcinoma of the body is recognized because of its yellowish, friable, cheesy appearance. It is liable to undergo squamous metaplasia. So a differential diagnosis from cancer of the cervix may be difficult especially if the tumour is growing downwards to involve the cervix. The removal of a polyp must be accompanied by thorough curettage, as carcinoma of the body and a polypus may co-exist.

The significant association with diabetes is interesting. Often it is only when the patient comes into hospital with post-menopausal bleeding that the diabetes is recognized.

### TREATMENT

Until quite recently if carcinoma of the body was found on curettage the uterus was packed and the cervix sutured and then bilateral salpingo-oöphorectomy and total hysterectomy carried out. With such treatment 70 per cent. of cases were alive at the end of five years. The response of this carcinoma to radium is not generally good even in early cases; and only Heymann in Stockholm has persisted in treating it entirely with radium. Recently, one radium treatment has been employed in order to cause necrosis of the superficial growth and to " freeze " any lymphatics prior to surgery and with this treatment it is hoped to avoid the implantation of secondaries in the vault. Whether this will in fact improve the results remains to be seen.

High-voltage therapy is reserved for recurrences, or inoperable cases.

### CANCER OF THE UTERUS—LATE STAGES

So often the general practitioner is left with a case at home which has either progressed too far for treatment or has recurred after treatment. If so he will perhaps be faced with a patient with incontinence of urine or faeces. Colostomy or transplantation of the ureters may be done as palliative measures. On the other hand he may have to treat a patient with severe bone pain which is worse at night and requires increasing dosage of morphia. Nowadays there are one or two long-acting morphia preparations for such cases, e.g. " Dromoran " (Roche). Some alleviation can be given by neurosurgeon or anaesthetist by injection of the involved nerve track with a long-acting local anaesthetic, e.g. " Proctocaine "; or the nerve may be destroyed at its root by intraspinal alcohol injection. These are palliative measures only but perhaps we can make fuller use of them. Blood transfusion for progressive anaemia will only delay the inevitable outcome.

And so to end. Our efforts, short of a cure for this dread disease, must be directed to prevention or at the worst to early recognition of it. Many of the extended surgical procedures and certainly of palliative surgery make me feel like Hamlet:

> " Yea, this solidity and compound mass,
> With tristful visage, as against the doom,
> Is thought-sick at the act." (Act III, Sc. iv.)

or again

> " But it reserv'd some quantity of choice
> To serve in such a difference."

I wish to record my thanks for the co-operation of my colleagues in the Radiotherapy Department, Dr. R. C. Tudway and Dr. S. Curwen, and Dr. A. L. Taylor (Pathology).

### APPENDIX

*University of Bristol Department of Obstetrics and Gynaecology*

### RESULTS 1951–1954

Total number of cases- 59
Cervix: 38
Body: 21

*Cervix*

| | |
|---|---|
| Radium only | 19 |
| Radium—Wertheim Hysterectomy | 17 |
| One case died without treatment | 1 |
| Radium and later partial exenteration "North America" (died at operation) | 1 |

Of 17 cases treated with radium and hysterectomy 7 had positive lymphatic nodes: 5 of these 7 cases are alive and well with no evidence of recurrence; 14 of the 17 cases survive and now have no evidence of recurrence. No case died as a result of Wertheim hysterectomy.

There were two cases of fistula: (*a*) vesico-vaginal (closed spontaneously); (*b*) uretero-vesico-vaginal (closed by surgery with re-implantation of ureter which is functioning satisfactorily).

Of the 19 cases treated by radium alone 10 survive, and of these 8 have no evidence of recurrence.

*Body*

14 treated with radium plus total hysterectomy and bilateral salpingo-oöphorectomy. (One died 5 days after operation of coronary disease—diabetic).
2 treated by total hysterectomy and bilateral salpingo-oöphorectomy.
3 treated with radium and H.V.T. (two now dead).
1 treated with radium and partial exenteration —"North America"—(died after eight months).
1 treated by total hysterectomy followed by H.V.T. (post-operative histological report of adenocarcinoma of polypoidal endometrium).
17 cases survive and none show evidence of recurrence so far.

### REFERENCES

*Annual Report from the South-Western Regional Cancer Records Bureau*, 1950.
Bonney, V. (1949). *Lancet*, **1**, 637.
Brunschwig, A. Twelfth British Congress of Obstetrics and Gynaecology, 137.
Currie, D. W. (1952). *Proceedings of the Royal Society of Medicine*, **45**, 327.
Glucksmann, A. Twelfth British Congress of Obstetrics and Gynaecology, 129.
Gourney, P. (1955). *Lancet*, **1**, 248.
Green-Armytage, V. B. (1950). *Proceedings of the Royal Society of Medicine*, **43**, 976.
Hertig, A. T. (1952). *American Journal of Obstetrics and Gynaecology*, **64**, 815.
Liu, W., & Meigs, J. V. (1955). *American Journal of Obstetrics and Gynaecology*, **64**, 815.
Randall, C. L. (1946). *Progress in Gynaecology*, Heinemann, London, 335.
Read, C. D. (1948). *Edinburgh Medical Journal*, **55**, 675.
Redman, T. F. (1952). *Proceedings of the Royal Society of Medicine*, **45**, 331.
Way, S. (1951). *Malignant Disease of the Female Genital Tract*, Churchill, London.
Way, S. (1954). *Journal of Obstetrics and Gynaecology of the British Empire*, **61**, 46.

## Figures and Tables

**Fig. 1. f1:**